# Evaluating the effects of galbanic acid on hydrogen peroxide-induced oxidative DNA damage in human lymphocytes

**Published:** 2014

**Authors:** Kobra Shirani, Javad Behravan, Fatemeh Mosaffa, Mehrdad Iranshahi, Babak Mehmankhah, Kamal Razavi-Azarkhiavi, Gholamreza Karimi

**Affiliations:** 1 *Department of Pharmacodynamy and Toxicology, Faculty of Pharmacy, Mashhad University of Medical Sciences, Mashhad, I. R. Iran*; 2 *Biotechnology Research Center and Pharmacy School, Mashhad University of Medical Sciences, Mashhad, I. R. Iran*; 3 *Department of Pharmacognosy and Biotechnology, Faculty of Pharmacy, Mashhad University of Medical Sciences, I. R. Iran*; 4 *Medical Toxicology Research Center and Pharmacy School, Mashhad University of Medical Sciences, Mashhad, I. R. Iran*

**Keywords:** *Cometassay*, *DNAdamage*, *Galbanicacid*, *Ferulaszowitsiana*

## Abstract

**Objective**: *Ferula szowitsiana* has been widely used for medicinal purposes around the world. The anti-oxidant effect of *F. szowitsiana *had been proved. The current study aims to determine the protective effects of galbanic acid, a sesquiterpene coumarin from *F. szowitsiana*, against hydrogen peroxide (H_2_O_2_) - induced oxidative DNA damage in human lymphocytes.

**Materials and Methods**: Human lymphocytes were incubated with H_2_O_2_ (0, 25, 50, 100, and 200 µM), galbanic acid (200 and 400 µM) and a combination of galbanic acid (200 and 400 µM) and H_2_O_2_ (25 µM) at 4 C for 30 minutes. Solvents of galbanic acid without H_2_O_2_ were used as negative controls.

**Results:** The findings of this study demonstrated that H_2_O_2_ exposure leads to a significant concentration-dependent increase in DNA damage. Galbanic acid did not cause DNA damage compared with the control cells. Data showed that galbanic acid does not have a protective effect against H_2_O_2_-induced oxidative DNA damage in human lymphocytes.

**Conclusion**: According to the results, it is concluded that the capability of *F. szowitsiana *in reducing reactive oxygen species and the anti-inflammatory property of its methanolic extract may be due to its other ingredients.

## Introduction

Oxidative stress, defined as a disturbance in the balance between the production of reactive oxygen species (free radicals) and antioxidant defenses. It may have a part in pathogenesis of various diseases, including cancer, diabetes, aging and other degenerative diseases (Uttara et al., 2009 ▶, Lobo et al., 2010 ▶). Antioxidants are substances capable of inhibiting or slowing the oxidation of other molecules. They are different in shape, physicochemical properties and their site of action (Flora 2009 ▶). Antioxidants can be natural or synthetic (Yeo et al., 2010 ▶). 

Some synthetic antioxidants such as butylated hydroxyanisole (BHA) and butylated hydroxytoluene (BHT) are believed to be responsible for liver damage and cancer in laboratory animals (Biswas, Haldar et al., 2010 ▶). As a result, there is renewed interest in the increased use of naturally occurring antioxidants. Because they occur in nature and in many cases are derived from plant sources, natural antioxidants are presumed to be safe (Ji et al., 2009 ▶). Phytochemicals are found in fruits, vegetables, and plant-derived beverages and may prevent some of the damage caused by free radicals (Stevenson and Hurst 2007 ▶, Lin et al., 2013 ▶). 

Several studies have been done on the antioxidant compounds from *Ferula* species (Gholitabar and Roshan 2013 ▶, Kavoosi and Rowshan 2013 ▶, Kavoosi et al., 2013 ▶). One of the most important compounds of *F. szowitsiana* are sesquiterpenes particularly sesquiterpene coumarins (Shahverdi et al., 2007 ▶, Bazzaz et al., 2010). Coumarins represent a wide spectrum of biological activity such as antibacterial, antifungal, anti-inflammatory, anticoagulant, anti-HIV and antitumor (Wu et al., 2009 ▶, Riveiro et al., 2010 ▶)**.** Galbanic acid is an isolated sesquiterpene coumarin from *Ferula* species with antibacterial, anti-tumor and anti-proliferation activities (Kim et al., 2011). The aim of this study was to assess the ability of galbanic acid in protecting human lymphocytes against H_2_O_2_- induced oxidative DNA damage.

## Materials and Methods

**Chemicals**

Low melting point agarose (LMA) and normal melting agarose (NMA) were purchased from Fermentas International, Inc. (Canada). All the remaining chemicals were obtained from Merck (Germany).

**Galbanic acid**

Galbanic acid was isolated from F. szowitsiana described previously (Iranshahi et al., 2007 ▶).

**Isolation of human lymphocytes**

Fasting blood was obtained from volunteer donors. Five milliliters of the whole blood was diluted 1:1 with PBS, and then carefully layered on top of a lymphocyte separation medium [aqueous solution of Ficoll, 57 g/L; density of 1.077 g/mL] in a centrifugation tube in a 1:1 ratio. After centrifugation for 20 minutes at 2000 rpm, gradient-separated lymphocytes were recovered, diluted 1:1 with PBS, and centrifuged a second time at 1500 rpm for 10 minutes. The cell pellets were resuspended in 500 mL of PBS, and the cells counted in a Neobauer chamber. The cell concentration was adjusted to 5000 cells/mL in preparation for the comet assay. Cell viability was determined using the trypan blue dye exclusion method, and only cell suspensions with viabilities of more than 96% were used for determination of DNA damage.

**Determination of DNA damage [comet assay]**

The comet assay was performed under alkaline conditions according to the technique described in 1988 (Singh et al., 1988 ▶). Human lymphocytes were incubated in different concentrations of H_2_O_2_ (50, 100, and 200 µM) as a positive control and different concentrations of galbanic acid (200 and 400 µM) alone. In the test groups, lymphocytes were exposed to H_2_O_2_ (25 µM) and 200 or 400 µM of galbanic acid at 4°C for 30 minutes. In addition, we used the galbanic acid solvents without H_2_O_2_ as negative controls.

Samples were then centrifuged at 3000 rpm for 10 minutes and the cells washed with PBS. The cell pellets were mixed with 100 mL of 0.75% (w/v) low melting point agarose (LMA), and then distributed onto microscope slides coated with 100 mL of 1% (w/v) normal melting agarose, covered with a cover slip, and kept for 10 minutes at 4°C to solidify. After the cover slips were removed, the slides were covered with another 100 mL of (0.75% w/v_ LMA, covered with a cover slip, and kept for 10 minutes at 4°C. After solidification, the slides were submerged in a cold fresh lysing solution [2.5 M NaCl, 100 mM Na_2_EDTA, 10 mM Tris, 1% (v/v) triton X-100, 10% DMSO, pH 10.0] for at least 2 hours. After the lysis, the slides were placed in an alkaline solution [1 mM Na_2_EDTA, 0.3 N NaOH, pH 13.0] for 40 min to permit unwinding of the DNA.

 Electrophoresis was run at 25 V for 45 minutes at 4°C. To prevent additional DNA damage all procedural steps were performed under yellow light conditions. The slides were then neutralized with 0.4 M Tris-HCl buffer, pH 7.5, stained with ethidium bromide (20 p.g/mL). The slides were studied using a fluorescent microscope (Nikonl00) connected to a CCD camera and a personal computer. Fifty individual cells were selected for calculations for each analysis, and four separate experiments [four slides for each experimental point] were conducted for each series. Single cells were analyzed with "Casp 1.2.2." software. The DNA damage was expressed as % Tail DNA, where % Tail DNA= [Tail DNA/ [Head DNA + Tail DNA]] x 100. A higher % Tail DNA indicated a higher level of DNA damage.

**Statistical analysis**

All statistical analyses were performed using SPSS analysis software (version 17.0) and the data are represented as mean ± SEM. ANOVA followed by Tukey test was used to compare the results obtained for the groups treated with galbanic acid and the negative control group. The difference was considered significant when p *value *was less than 0.05.

## Results

**Figure1:**
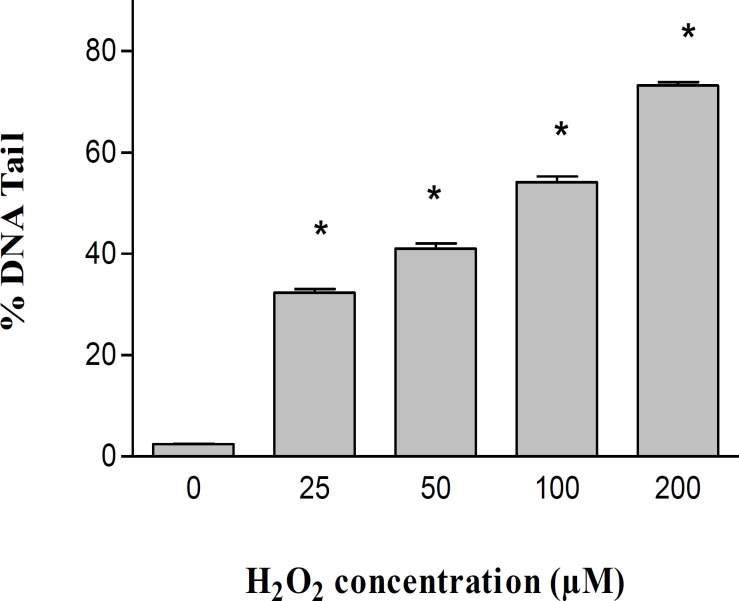
Level of DNA damage in H_2_O_2_-treated human lymphocytes. Lymphocytes were incubated for 30 minutes at 4°C with different concentrations of H_2_O_2_. Results are presented as mean ± SEM (n=4 slides × 50 lymphocytes). ANOVA was used for comparison and *p value* <0.05 was considered as significant and marked

**Figure 2:**
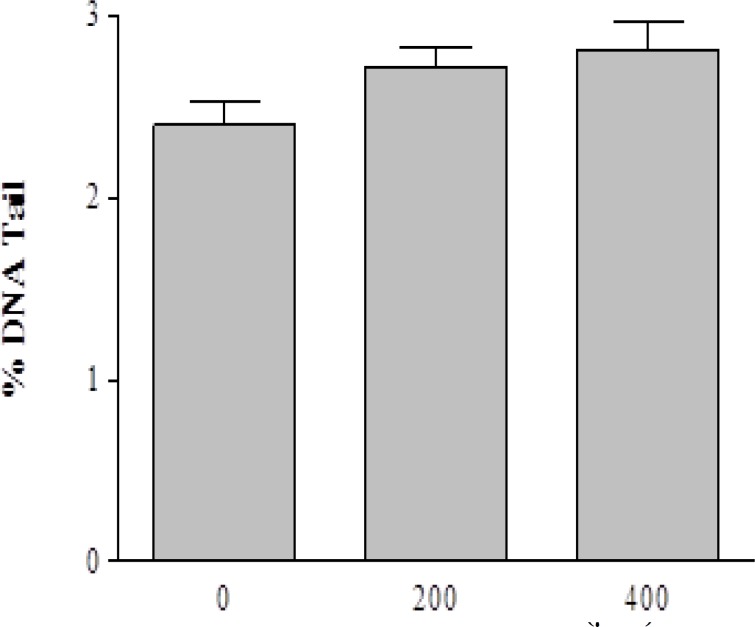
Level of DNA damage in human lymphocytes upon treatment with different concentrations of galbanic acid compare to negative control. Lymphocytes were incubated for 30 minutes at 4°C with galbanic acid. Results are mean±SEM (n=4 slides × 50 lymphocytes). ANOVA was used for group comparison in significance level of *p value* <0.05.

Data showed that H_2_O_2_ exposure (0, 25, 50, 100, and 200 µM) leads to a significant concentration-dependent increase in DNA damage when compared to control cells (Figure 1). Galbanic acid (200 and 400 µM) showed no toxicity against human lymphocytes (Figure 2).

**Figure 3:**
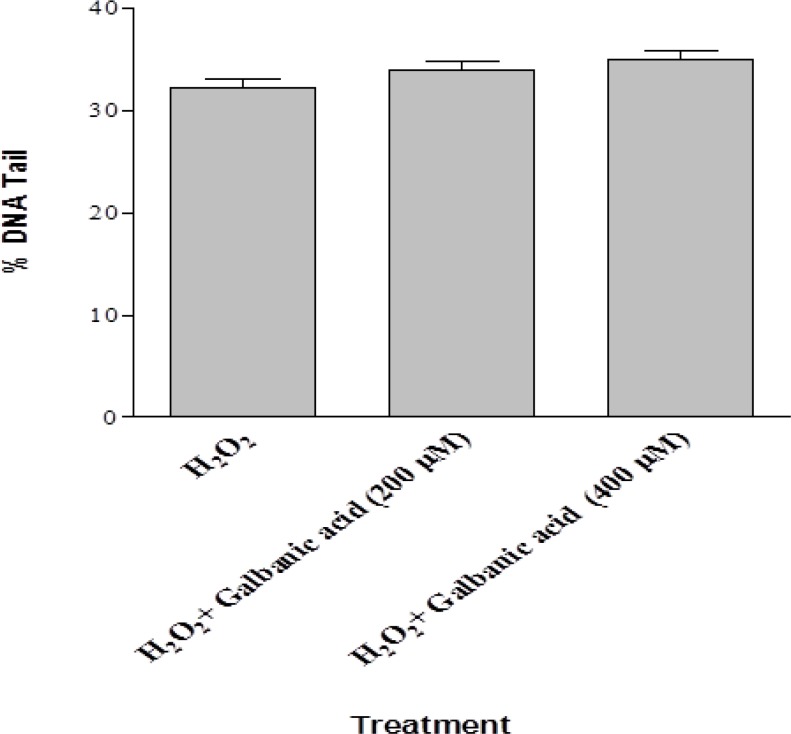
Effect of galbanic acid on lymphocyte DNA damage induced by H_2_O_2_. Human lymphocytes were incubated for 30 minutes at 4°C with a combination of H_2_O_2_ (25 µM) with different concentrations of Galbanic acid 200 and 400 µM). Results are mean±SEM (n=4 slides × 50 lymphocytes). ANOVA was used for group comparison in significance level of *p value* <0.05.

The data obtained from DNA damage in lymphocytes exposed to H_2_O_2_ (25 µM) and galbanic acid (200 and 400 µM) revealed that H_2_O_2_-induced DNA damage was unaffected due to galbanic acid treatment (Figure3)

## Discussion

The genus Ferula belongs to the Umbelliferae family with approximately 130 species distributed throughout the Mediterranean area and central Asia where many species have been traditionally used in folk medicine (Kim et al., 2011). F. szowitsiana is one of the ethnomedicinal plants in the genus that is widely used in Azerbaijan, Iran, Turkey and Afghanistan. Many studies reported a wide range of pharmacological effects of the plant, including anti-cancer, anti-inflammation, antimicrobial and antibiotic activities (Gholami et al., 2013 ▶, Paydar et al., 2013 ▶). Galbanic acid as an ingredient of F. szowitsiana, showed various biological properties, including anticancer, cancer chemopreventive, anticoagulant, antiviral, and antileishmanial activities (Kasaian et al., 2013 ▶). In this study we used the comet assay to find if this compound has any protective effect against H2O2-induced oxidative DNA damage in human lymphocytes.

The capability of F. szowitsiana in reducing ROS and its anti-inflammation property had been recognized. Soltani et al demonstrated antioxidant capacity in auraptene isolated from F. szowitsiana. Auraptene was proved to be more effective than ascorbic acid to decrease genotoxicity of H2O2. They suggested that prenyl moiety in auraptene may suppress the generation of superoxide anion (Soltani et al., 2010 ▶).

In another study, gooshchi et al evaluated antioxidant activity of n-hexan, dichloromethane and methanolic extracts of F. szowitsiana by 2, 2-diphenyl para-1-picryl hydrazyl (DPPH) method. The methanolic extract has shown significant antioxidant activity which was comparable with rutin. They demonstrated that F.szowitsiana is a rich source of coumarin and furanocomarin derivatives and these compounds may pose potent antioxidant property (Gooshchi et al., 2012). According to our results, it is concluded that galbanic acid does not have a protective effect against H2O2-induced oxidative DNA damage in human lymphocytes at the tested concentrations. The lack of efficacy may be due to the use of inappropriate concentrations. Therefore, the antioxidant properties of the methanolic extract of F. szowitsiana may be due to other compounds such as: umbelliferon-7-apiosyl β (16) glucoside, p-hydroxy phenylethanoid glucoside, umbelliferon β-D [6' (ferolyle)-glucoside] and umbelliferon. Further research should be done to investigate the antioxidant activity and antigenotoxic effects of these compounds (Gooshchi et al., 2012).
